# Added prognostic value of molecular imaging parameters over proliferation index in typical lung carcinoid: an [18F]FDG PET/CT and SSTR imaging study

**DOI:** 10.1007/s12149-022-01797-7

**Published:** 2022-10-30

**Authors:** Flavia Linguanti, Elisabetta M. Abenavoli, Vittorio Briganti, Ginevra Danti, Daniele Lavacchi, Maria Matteini, Luca Vaggelli, Luca Novelli, Anna M. Grosso, Francesco Mungai, Enrico Mini, Lorenzo Antonuzzo, Vittorio Miele, Roberto Sciagrà, Valentina Berti

**Affiliations:** 1 Nuclear Medicine Unit, Department of Experimental and Clinical Biomedical Sciences ``Mario Serio’’, University of Florence, 50134 Florence, Italy; 2 Nuclear Medicine Unit, Careggi University Hospital, Largo Brambilla 3, 50134 Florence, Italy; 3 Department of Radiology, Careggi University Hospital, Largo Brambilla 3, 50134 Florence, Italy; 4 Unit of Pneumology and Thoracic-Pulmonary Physiopathology, Careggi University Hospital, Largo Brambilla 3, 50134 Florence, Italy; 5 Medical Oncology Unit, Careggi University Hospital, Largo Brambilla 3, 50134 Florence, Italy; 6 Department of Experimental and Clinical Medicine, University of Florence, Largo Brambilla 3, 50134 Florence, Italy; 7 Department of Pathology, Careggi University Hospital, Largo Brambilla 3, 50134 Florence, Italy; 8 Department of Health Sciences, University of Florence, 50139 Florence, Italy

**Keywords:** Typical lung carcinoid, Prognosis, PET, Ki67

## Abstract

**Objective:**

This study was performed to evaluate the prognostic meaning of volumetric and semi-quantitative parameters measured using [18F]FDG PET/CT and somatostatin receptor (SSTR) imaging in patients with typical lung carcinoid (TC), and their relationship with proliferative index (Ki67).

**Methods:**

We retrospectively reviewed 67 patients (38–94 years old, mean: 69.7) with diagnosis of TC who underwent [18F]FDG PET/CT and/or SSTR scintigraphy/SPECT with [111In]DTPA-Octreotide plus contrast-enhanced CT (CECT) at staging evaluation. All patients had Ki67 measured and a follow-up (FU) of at least 1 year. SSTR density (SSTRd) was calculated as the percentage difference of tumor/non-tumor ratio at 4 and 24 h post-injection. At PET/CT, metabolic activity was measured using SUVmax and SUVratio; volumetric parameters included MTV and TLG of the primary tumor, measured using the threshold SUV41%. ROC analysis, discriminant analysis and Kaplan–Meier curves (KM) were performed.

**Results:**

11 patients died during FU. Disease stage (localized versus advanced), SUVratio, SUVmax, Ki67, MTV and TLG were significantly higher in non-survivors than in survivors. ROC curves resulted statistically significant for Ki67, SUVratio, SUVmax, MTV and TLG. On multivariate analysis, stage of disease and TLG were significant independent predictors of overall survival (OS). In KM curves, the combination of disease stage and TLG identified four groups with significantly different outcomes (*p* < 0.005). Metabolic activity (SUVmax and SUVratio) was confirmed as significant independent prognostic factor for OS also in patients with advanced disease, with the best AUC using SUVmax.

In patients with advanced and localized disease, SSTRd proved to be the best imaging prognostic factor for progression and for disease-free survival (DFS), respectively. In localized disease, SSTRd 31.5% identified two subgroups of patients with significant different DFS distribution and in advanced disease, a high cutoff value (58.5%) was a significant predictor of adverse prognosis.

**Conclusion:**

Volumetric and semi-quantitative parameters measured using [18F]FDG PET/CT and SSTR imaging combined with Ki67 may provide a reference for prognosis evaluation of patients with TC, to better stratify risk groups with the goal of developing individualized therapeutic strategies.

## Introduction

Neuroendocrine neoplasms (NENs) constitute a heterogeneous group of tumors that stem from cells of the neuroendocrine system. More than 25% of NENs arise within the bronchopulmonary system, reflecting the high density of Kulchitsky cells in the respiratory epithelium [1–4]. Typical carcinoid (TC) and atypical carcinoids (AC) represent a group of well-differentiated (WD) lung NENs, and among them, TC accounts for only 1–2% of all lung NENs. [5–7]. According to the WHO classification of 2015 TC have carcinoid morphology, less than 2 mitoses per 2 mm^2^ and lacking necrosis, whereas atypical carcinoid tumors (ACs) have carcinoid morphology with 2–10 mitoses per 2 mm^2^ or necrosis (often punctate) [8, 9]. Ki67 index is a marker of cell proliferation that it is incorporated into the 3-tiered grading system for NENs of the gastrointestinal and pancreaticobiliary tract [10, 11]. However, it is not currently used in the WHO grading scheme for lung NENs, except to separate the high-grade small cell lung carcinoma (SCLC) and large cell neuroendocrine carcinoma (LCNEC) from the WD [12–16].

Although TC is considered tumors with indolent behavior, within the group, there is a great variability in clinical outcome [17, 18]. Therefore, early and specific diagnosis is important. Currently, contrast-enhanced computed tomography (CECT), somatostatin receptor scintigraphy with [^111^In-DTPA-D-Phe^l^]-octreotide ([111In]DTPA-octreotide) (Octreoscan®) and positron emission tomography/computed tomography (PET/CT) with either [68 Ga]DOTA-peptides or 2-deoxy-2-[18F]fluoro-D-glucose ([18F]FDG) are the most widely used imaging modalities for the localization and characterization of TC [19–22]. Based on the presence of somatostatin receptors (SSTR) on the surface of lung TC cells, the use of radiolabeled somatostatin receptor analogues (such as [^111^In]DTPA-octreotide and [68 Ga]DOTA-peptides) plays a crucial role in the evaluation of SSTR density (SSTRd) in patients with TC [23–26]. Conversely, [18F]FDG PET is most used in aggressive tumors with high Ki67 indices and lower expression of SSTR [27–30]. The most used parameter for the quantification of metabolic activity is standardized uptake value (SUV) [31, 32]. Recently, other metabolic parameters such as metabolic tumor volume (MTV) and total lesion glycolysis (TLG) have been introduced as more comprehensive parameters that better reflect metabolic tumor burden than SUVmax [33–35].

To the best of our knowledge, there are no reports in the literature on the correlation between nuclear medicine imaging features and Ki67 in lung TC. In our retrospective study, we aimed to evaluate the prognostic meaning of volumetric and semi-quantitative parameters measured using [18F]FDG PET/CT and SSTR imaging in patients with TC, and their added value to Ki67 in determining the patient outcome.

## Materials and methods

### Patients and data collection

Sixty-seven patients with histological diagnosis of pulmonary TC, who at staging underwent nuclear imaging ([18F]FDG PET/CT and/or SSTR scintigraphy/single-photon emission computed tomography (SPECT) with [111In]DTPA-octreotide) and CECT in our Institution were included in a retrospective study. This study was approved by our Institutional Ethics Review Board (protocol number 14776). All patients provided written informed consent and their images were anonymized and de-identified before analysis.

All patients with histologically proven lung TC diagnosed between January 2009 and June 2019 were selected retrospectively and classified according to the current WHO classification [5]. The histopathological features considered for TC grading were the mitotic count and the presence or absence of necrosis [8]. An additional histopathological parameter is Ki67 index that is required for an adequate diagnosis of TC. The clinical or pathological stage was performed based on the Tumor-Node-Metastasis (TNM) staging system for Lung cancer following the American Joint Committee on Cancer Guidelines (AJCC), 8th edition [36]. Patients were divided in two subgroups: patients with localized disease (stage I–II) and patients with advanced disease (stage III–IV) according to previous AJCC guidelines [36]. All patients had a clinical follow-up of at least 1 year (range 12–139 months).

### Somatostatin receptor scintigraphy imaging

Patients were well hydrated before and for at least 1 day after injection. Patients were injected with 222 MBq (6 mCi) of [111In]DTPA-octreotide, a [111In]DTPA-D-Phe- conjugate of octreotide and a long-acting somatostatin analogue. Image acquisition was performed on a Siemens E.CAM 6940 or a Siemens E.CAM Duet 8457 using a large field of view (FOV) gamma camera fitted with a medium-energy collimator. Symmetrical 20% energy windows were centered over both photopeaks of 111In (173 and 247 keV), adding the data from both windows. Whole-body scan (WB) and SPECT imaging were performed 4 and 24 h after injection according to the following parameters: WB scan for 20 cm/min using 256 × 256 matrix and SPECT images of the appropriate regions using 64 × 64 matrix, 360° rotation, 40 s/frame.

### [18F]FDG PET/CT

All patients fasted for at least 4 h before examination. Images were obtained from skull base to mid-thigh on a dedicated PET/CT scanner (Philips Gemini TF 16 PET/CT), 60 min after intravenous injection of 3.6 MBq/kg of FDG. CT acquisition (120 kV, 30–150 mAs) was performed on spiral 16 slice CT with a slice thickness of 4 mm. After transmission scan, 3D PET acquisition was taken for 2 min/field with an axial FOV of 57.6 cm. CT-based attenuation correction of the emission images was performed. PET images were reconstructed by the iterative method. After completion of PET acquisition, the reconstructed attenuation corrected PET images, CT images and fused images of matching pairs of PET and CT images were available for review in axial, coronal and sagittal planes and maximum intensity projections (MIPs).

### CECT imaging

All CT investigations were carried out on a 64-detector helical CT (Somatom Sensation 64). A standard protocol was used; the patients were scanned in supine with craniocaudal breath-hold scans; all cases underwent non-contrast and CECT. Iodinated contrast medium (Ultravist 370) was injected in the antecubital vein at the flow rate of 3–4 mL/using an automatic injector, immediately followed by a saline flush (40–50 mL) with a rate of injection of 3–4 mL/s. The dose of contrast medium was administered according to the patient body weight (mL/kg body weight: 80–100 mL (< 80 kg) or 100–120 mL (> 80 kg)). Dual-phasic contrast-enhanced images were obtained during the arterial phase (30–35 s after the start of injection) and portal venous phase (70–75 s after the initiation of the injection). The parameters for both non-contrast and contrast-enhanced CT examination were tube voltage, 120 kV; tube current, 200–250 mAs, depending on the patient’s size; beam collimation, 64 × 0.5 mm; rotation time, 0.4 s; pitch, 1; and reconstruction interval, 1 mm.

### Evaluation of images

Two expert radiologists (> 10 years of practice) retrospectively analyzed CECT aspects of all TC patients. It was evaluated the size of the lesion, in particular the major axis. Nuclear images were retrospectively evaluated by two nuclear medicine specialists, with 10 and 30 years of experience, respectively. SSTRd was measured as the tumor/non-tumor ratio at each time point.

Tumor uptake was measured by designing a manual region of interest (ROI) in the primary lung lesion. Non-tumor uptake was measured using a ROI of the same dimension of the previous one but positioned on the contralateral lung. SSTRd was then estimated as the ratio between the tumor/non-tumor uptake (TNT) difference at the two time points (24 h–4 h) and the 4-h value, × 100 [37]. High percentage values were defined as elevated SSTRd.

We measured the SUV of malignant lesions by drawing a ROI on axial plan PET for the semi-quantitative analysis. The ROI was placed over the area of maximum activity within the malignant lesion, and the SUVmax was determined as the highest SUV of the pixels within the ROI. Then, FDG uptake was measured as the ratio between lesion SUVmax and liver SUVmean (SUV ratio). SUV is defined as: lesion ROI uptake (MBq/ml)/[injected dose (MBq)/body weight (kg)].

**Fig.1:**
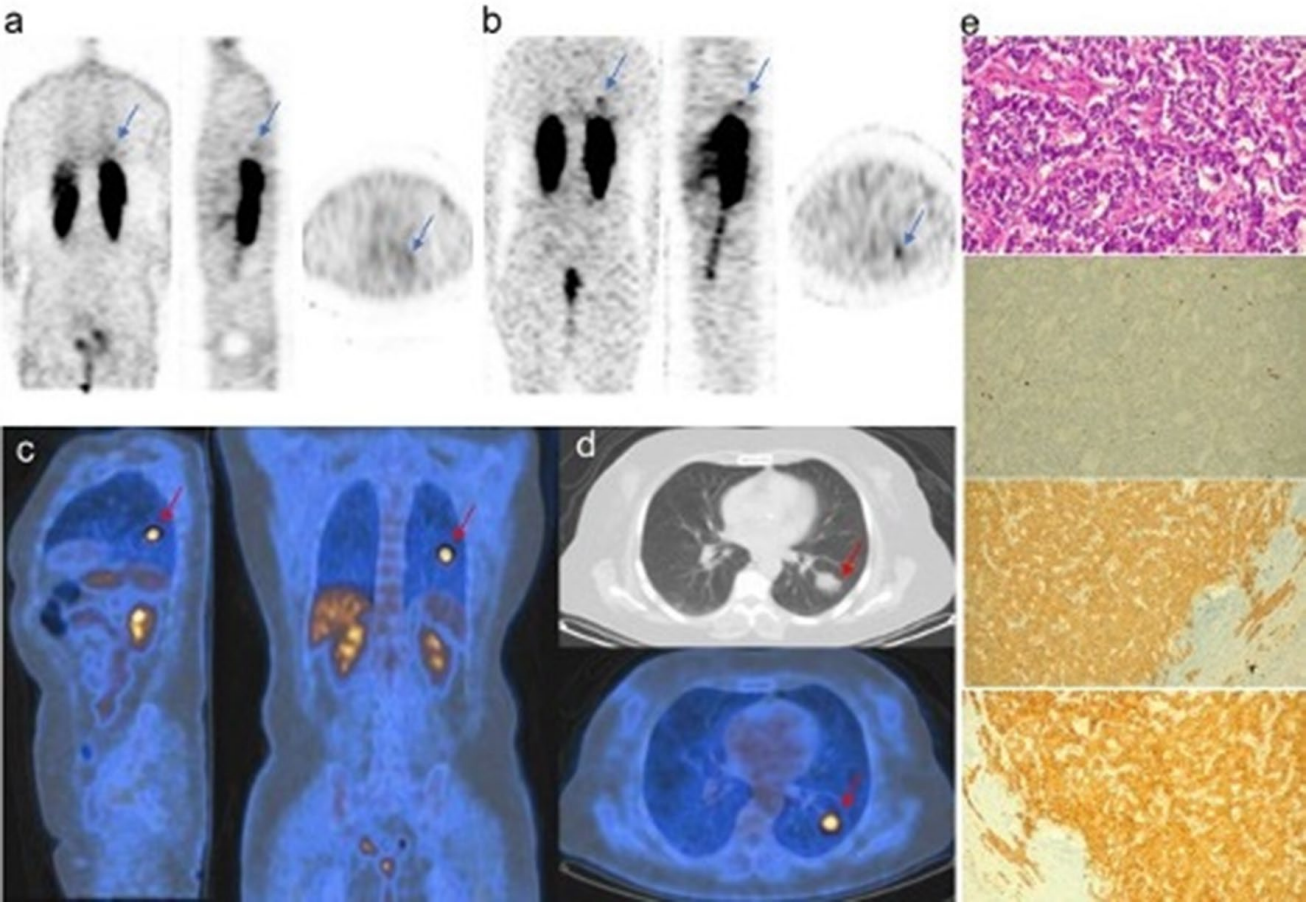
Example of typical carcinoid patient who underwent at staging [18F]FDG PET/CT, SSTR scintigraphy/SPECT with [111In]DTPA-octreotide, CECT and histopathological analysis. A 64-year-old female with typical carcinoid of the left lung, who underwent to Octreoscan SPECT at 4 h (**a**) and 24 h (**b**),18F-FDG PET/CT (**c**) and CECT (**d**). Parameters: SSTRd 53%. SUVmax 4.6. SUVratio 1.80. MTV 5.4 cm^3^ and TLG 16.65 g. Size 22 mm. Anatomo-pathological features (**e**): typical carcinoid. ChromograninA +, Synaptophysin +,Hematoxylin Eosin: absence of necrosis, mitoses < 2/2 mm^2^ and ki67 < 5%)

We also measured the MTV and TLG of each lesion using a semiautomatic method to delineate the volume of interest (VOI) over the malignant lesion. We used a SUV-based contouring software (FIJI software ImageJ 2011 version) [38]. A SUV threshold value of 41% of the SUVmax was used [31]. The program also analyzed the SUVmean of each lesion, and the TLG value was calculated by multiplying MTV by SUVmean. Finally, the expert pathologist (20 years of practice) provided Ki67 index, expressed as a percentage, for all the lung TC patients included in the study (Fig. 1)**.**

### Statistical analysis

All statistical analyses were performed using IBM SPSS Statistics software (version 25.0). Comparison between groups was performed using one-way ANOVA or two-sample T test, when appropriate. Analysis of prognostic factors was carried out using receiver-operating curves (ROC), Cox regression analysis and Kaplan–Meier method with Log rank test. A *p* value < 0.05 was considered significant.

## Results

Eighty-three patients with histological diagnosis of lung TC between January 2009 and June 2019 were selected. Sixteen patients did not undergo staging CECT and/or Octreoscan SPECT and [18F]FDG PET and were excluded. Thus, the study cohort included 67 patients. Demographical and clinical data are summarized in Table 1. Furthermore, imaging data are reported in Table 2.

**Table 1:** Demographic and clinical data of the study population

Characteristic	
Gender, *n* (%)	
Male	24 (35.8%)
Female	43 (64.1%)
Age, mean y (range)	69.7 (38–94)
Stage, *n* (%)	
I	42 (62.7%)
II	8 (11.9%)
III	5 (7.4%)
IV	12 (17.9%)
Localized	50 (74.6)
Advanced	17 (25.4)
Ki67, *n* (%)	
≤ 2	14 (20.9%)
2–10	38 (58.2%)
> 20	14 (20.9%)
Treatment, *n* (%)	
Surgery	50 (74.6%)
Somatostatin analogue	10 (14.9%)
Chemotherapy	5 (7.4%)
Follow-up	2 (2.9%)
Follow-up	
Months, mean (range)	67 (12–139)
Survivors, *n* (%)	55 (82.1%)
Non-survivors, *n* (%)	12 (17.9%)
Imaging, *n* (%)	
CECT	67 (100%)
111In-Octreoscan	12 (17.9%)
18F FDG PET/CT	28 (41.8%)
111In-Octreoscan/18F FDG PET/CT	27 (40.3%)

*CECT* contrast-enhanced computed tomography, *FDG* *PET/CT* fluorodeoxyglucose positron emission tomography/computed tomography, *SSTRd* somatostatin receptor density

**Table 2:** Distribution of the different examinations according to the patient groups

	111In-Octreoscan (*n* = 12)	18F FDG PET (*n* = 28)	111In-Octreoscan/18F FDG PET (*n* = 27)	SSTR density (*n* = 39)	Metabolic activity (*n* = 55)
Localized	11 (91.6%)	22 (78.6%)	17 (62.9%)	28 (71.8%)	39 (70.9%)
Advanced	1 (8.3%)	6 (21.4%)	10 (37.1%)	11 (28.2%)	16 (29.1%)
Survived	11 (91.6%)	21 (75%)	23 (85.2%)	34 (87.2%)	44 (80%)
Not survived	1 (8.3%)	7 (25%)	4 (14.8%)	5 (12.8%)	11 (20%)
Ki67 ≤ 2	4 (33.3%)	5 (17.8%)	5 (18.5%)	9 (23.1%)	10 (18.2%)
Ki67 2–10	7 (58.3%)	15 (53.6%)	17 (62.9%)	24 (61.5%)	32 (58.2%)
Ki67 > 10	1 (8.3%)	8 (28.6%)	5 (18.5%)	6 (15.4%)	13 (23.6%)

FDG PET/CT fluorodeoxyglucose positron emission tomography/computed tomography; SSTRd somatostatin receptor density; Ki67 proliferative index

Analyzing patients according to OS, there was a significant difference between survivors and not-survivors in disease stage (localized versus advanced), Ki67, SUVmax, SUVratio, MTV and TLG, but not in CT dimensions and SSTRd (*p* < 0.01). Mean values and SD of all variables are reported in Table 3. Among these significant variables, ROC curve analysis found the largest area under the curve (AUC) for TLG (AUC = 0.927), followed by SUVratio (AUC = 0.897), SUVmax (AUC = 0.848), MTV (AUC = 0.846) and Ki67 (AUC = 0.810), the smallest for SSTRd (AUC = 0.671). These results were confirmed when the analysis was performed in the subgroup of 27 patients who underwent both [18F] FDG PET and Octreoscan imaging. ROC curve analysis found the largest AUC for TLG (AUC = 0.988), followed by MTV (AUC = 0.938), SUVratio (AUC 0.862), and SUVmax (AUC = 0.750), and the smallest for SSTRd (AUC = 0.256).

**Table 3:** Summary of all analyzed variables

	OS (0 = alive, 1 = died)	Mean ± *SD*	*p*
Size	0	26.15 ± 17.1	0.415
	1	28.25 ± 10.7	
SSTRd	0	74.02 ± 36.2	0.841
	1	53.20 ± 36.2	
SUVratio	0	1.14 ± 0.5	< 0.001
	1	2.60 ± 1.1	
SUVmax	0	2.81 ± 1.3	< 0.001
	1	5.87 ± 2.9	
MTV41%	0	12.34 ± 20.2	< 0.001
	1	219.03 ± 353.5	
TLG41%	0	31.31 ± 75.6	< 0.001
	1	856.10 ± 1429.1	
Ki67%	0	4.49 ± 5.0	< 0.001
	1	15.87 ± 15.2	

*OS* overall survival, *SD* standard deviation, *SSTRd* somatostatin receptor density, *SUVmax* maximum standard uptake value, *MTV* metabolic tumor volume (threshold 41% of SUVmax lesion), *TLG* total lesion glycolysis (threshold 41% of SUVmax lesion), *Ki67%* proliferative index

**Fig. 2:**
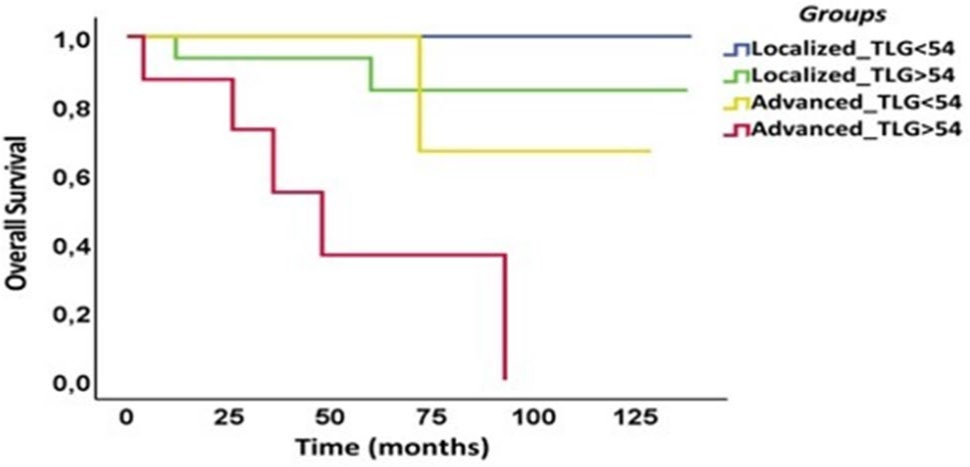
Kaplan–Meier curves of overall survival in the patient population groups identified by Disease Stage and TLG. At Kaplan–Meier curves, combining disease stage (localized versus advanced) with TLG (total lesion glycolysis), the population was divided into 4 groups (*p* < 0.005). The first group identified patients with localized disease and low TLG (< 54), the second group those with localized disease and high TLG (> 54), the third and fourth groups those with advanced stage and TLG, respectively, below or above the cutoff

The optimal cutoff values were: TLG = 54 g and Ki67 = 7.5%. Disease stage (*p* < 0.0001), TLG (*p* < 0.002) and Ki67 (*p* < 0.003) were identified as significant predictors of OS. On multivariate analysis, disease stage (HR = 0.072, 95% CI 0.14–0.358) was a significant independent predictor of OS along with TLG (HR = 0–089 95% CI = 0.11–0.733). Kaplan–Meier survival curves were generated and were significantly different for stage (*p* = 0.0001), TLG (*p* < 0.003) and Ki67 (*p* = < 0.005). Then, we combined stage and TLG, thus dividing the population into 4 groups: the first group identified patients with localized disease and low TLG (< 54), the second group those with localized disease and high TLG (> 54), the third and fourth groups those with advanced stage and TLG, respectively, below or above the cutoff. The survival distribution among the groups resulted significantly different (*p* < 0.005) (Fig. 2).

### Patients with localized disease

Among patients with localized disease, there were just two deaths during follow-up, so no separate OS analysis was performed. In these patients, there was a significant difference between those with disease-free survival (DFS) and the other subjects in CT dimensions, SSTRd, MTV and TLG (*p* < 0.05), but not in Ki67, SUVmax, SUVratio and type of treatment. The largest AUC was obtained using SSTRd (AUC = 0.875), followed by Ki67 (AUC = 0.767). The optimal cutoff values for identifying DFS in patients with localized disease were Ki67 = 2.5% and SSTRd = 31.5%, but only SSTRd was identified as significant predictor of DFS at regression analysis (*p* < 0.002).

Using the same SSTRd cutoff, a log rank test was performed to establish whether there were differences in the DFS distribution for the different SSTRd values. The survival distributions for the two groups of values were significantly different, χ^2^ = 5.25, p < 0.02. In this analysis, however, the combination of Ki67 and SSTRd according to their respective cutoffs did not allow to classify patient subgroups with different outcome.

### Patients with advanced disease

**Fig. 3:**
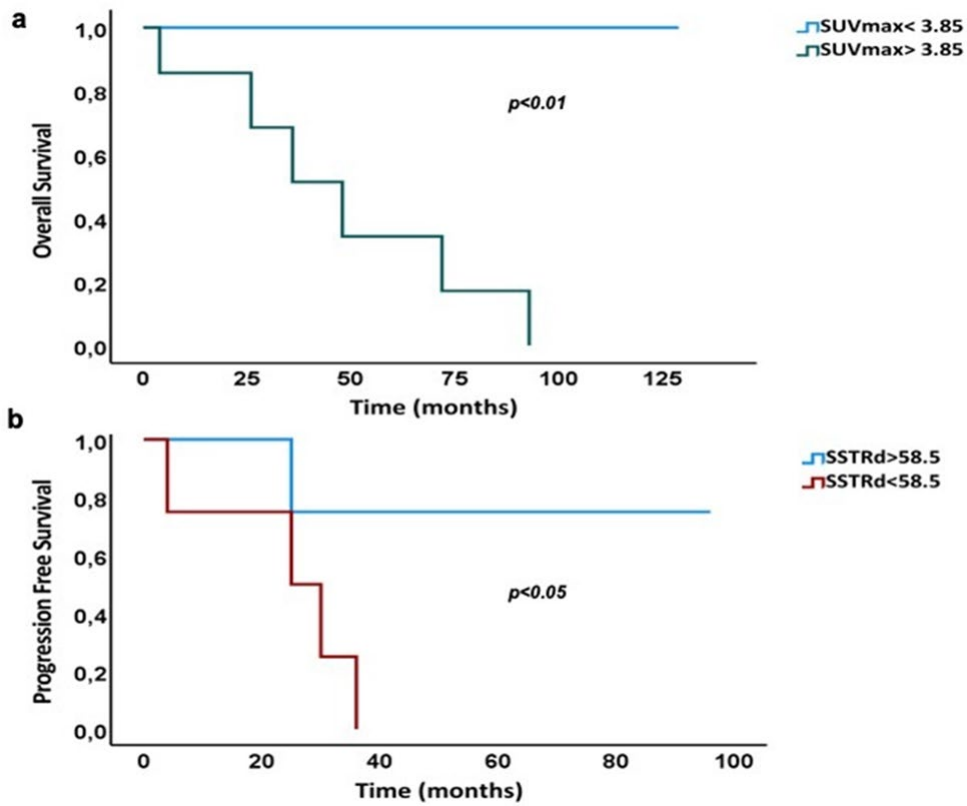
Kaplan–Meier curves of PFS and OS in patients with advanced disease. Kaplan–Meier survival curves comparing (**a**) overall survival (OS) grouped by the cutoff SUVmax 3.85; and (**b**) progression-free survival (PFS) grouped by the cutoff SSTRd 58.5

Dividing the patients with advanced disease according to their OS, there was a significant difference between survivors and not-survivors in SUVmax and SUVratio (*p* < 0.01), but not in the other variables, including treatment approaches. For OS, the best AUC was obtained using SUVmax (AUC = 0.986). The optimal cutoff value in advanced disease was SUVmax at 3.85, and it was identified as significant predictor of OS (*p* < 0.01) (Fig. 3a).

Using the cutoff of Ki67 at 7.5%, the survival distribution for the two derived groups was not significantly different. Analyzing patients according to Progression Free Survivals (PFS), there was a significant difference between the two groups in Ki67, SSTRd according to their respective cutoffs, and a borderline difference in SUVratio, (*p* < 0.05), but not in the other variables. No significant difference was observed among treatments. For PFS the best AUC was obtained using SSTRd (AUC = 0.933), followed by Ki67 (AUC = 0.833). Again, using the cutoff of Ki67 at 7.5%, the survival distributions for the two groups were not significantly different. SSTRd, using cutoff 58.5, was a significant predictor of adverse prognosis, identifying significantly different PFS curves (*p* < 0.05) (Fig. 3b). SSTRd and Ki67 together identified 3 groups with significantly different PFS curves (*p* < 0.01): first and second groups with low Ki67 and, respectively, high SSTRd or low SSTRd, and third group with high Ki67/low SSTRd.

## Discussion

Although TC is in general considered tumors with indolent behavior, there is nevertheless a great variability in clinical outcome within the affected patients and early diagnosis and proper staging are important. Surgical resection is the mainstay treatment for lung TC; in those with advanced disease, treatment may include somatostatin analogues therapy (SSA), targeted therapy, chemotherapy (CHT) or peptide receptor radionuclide therapy (PRRT) [16].

Until now, disease stage, which is the most important prognostic factor, and other prognostic factors, such as proliferation index Ki67, may be used as surrogates for the underlying tumor burden, which is a direct predictor of disease progression and survival [39]. In this study, we assessed the added prognostic role of several imaging biomarkers in addition to disease stage and Ki67.

As expected, patients with higher/advanced disease stage have a worse outcome, with an average OS of 79 months, as compared to patients with localized disease, with an average OS of 133 months. Ki67 and TLG were identified as significant independent prognostic factors for OS. This result is in line with the literature [40]. However, at a multivariate analysis in the whole group of patients, only stage and TLG together were able to predict patient OS. These results suggest that functional imaging with [18F]FDG PET provides metabolic, volumetric and quantitative information, making it possible to more accurately depict tumor burden, and therefore better predict patient survival. This confirms the report by Pasquali et al. [27], who using [18F]FDG PET showed high metabolic activity in the patients with rapidly growing NENs or aggressive NENs with distant metastases. The amount of [18F]FDG uptake reflected the malignant behavior and the ability of NENs to grow rapidly, indicating a worse prognosis, as confirmed by Kubota [41].

Metabolic activity was confirmed as significant independent prognostic factor for OS in patients with advanced disease as well. The most significant imaging variable resulted SUVmax, performing even better than Ki67 in predicting OS. In our study, positive [18F]FDG PET scan is predictive of high aggressiveness and lower survival rate (with an average OS of 57 months for FDG positive, and 70 months for FDG negative). Although not commonly used in TC in the past, [18F]FDG PET is gaining importance even in NENs [28]. In the literature, several studies analyzed the diagnostic role of SUVmax in carcinoids, and they showed increased FDG uptake and malignant potential in some TC [42–44].

In agreement with previous studies, although the diagnostic sensitivity for TC detection of [18F]FDG PET is lower than SSTR imaging, we found 32 FDG positive lesions (SUVmax > 3) out of 67 TC. Our data demonstrate that also well-differentiated TC could have heterogeneous behavior, which may reflect primarily the biology of the tumor itself, but most importantly affects its prognosis. TC is often slow growing, but outcomes are variable, and metabolic activity seems to be the best prognostic factor.

Especially in advanced disease stage, we noted that Ki67 was no longer a significant predictor of prognosis, in contrast with other neoplasms [45–48]. Such result could reflect the wide differences in tumor biology between lung NENs, especially well-differentiated ones such as TC, and other lung cancers originating from other cells.

Beyond OS, we tested the prognostic value for PFS and DFS of the imaging biomarker in addition to Ki67. Both in patients with advanced and localized disease stage, SSTRd proved to be the best imaging prognostic factor for PFS and DFS, respectively. This could suggest that NENs differentiation could be more important for PFS and DFS than Ki67. The most widely used imaging modalities for SSTR imaging are Octreoscan SPECT or PET/CT with [68 Ga]DOTA peptide. In this study we used [111In]DTPA-octreotide, which is known to have a sensitivity of ∼90% for NET diagnostics [49–51]. To quantitate specific tracer binding to TC tissue, we used a simple semi-quantitative method (SSTRd) [37] The prognostic role of SSTRd in localized stage is in addition to the value of Ki67; among the subjects with high Ki67, only those with low SSTRd have a bad DFS, confirming its added prognostic value over Ki67. The same behavior was seen in advanced disease: high SSTRd values resulted the most significant independent predictor of PFS. SSTRd was not a significant prognostic factor for OS, as it was for PFS. It confirms that tumor aggressiveness, which is strictly related to patient survival, is better measured by metabolic activity [27].

The recent study of Zidan et al. [52] demonstrated a wide range of intra and inter-patient heterogeneity on receptor and metabolic nuclear imaging. Accordingly, our study suggests that an accurate preoperative staging of TC with integrated imaging (metabolic and receptor imaging), may allow better characterization; furthermore, by demonstrating the prognostic value of integrated imaging in our cohort of patients, we suggest its use for stratifying patients and selecting their optimal treatment. It would be desirable to validate these results on large patient populations.

The present study has several limitations. First, it is retrospective. Moreover, our population was small, and had rather heterogeneous treatments. Only 27 patients underwent both [18F]FDG PET and Octreoscan imaging, although our results were confirmed in the analysis performed in both patient subgroups. Finally, another limitation could be the use of SRS to evaluate SSTRd. The actual gold standard is PET/CT with [68 Ga]DOTA-peptides, but our study is retrospective, and [68 Ga]DOTA PET/TC was still not available at that time. Anyway, SPECT with [111In]DTPA-Octreotide had a good accuracy and offers as well the possibility of quantifying SSTRd [37].

In conclusion, our results suggest that both SSTR and metabolic imaging offer several parameters that could be effective prognostic factors, in addition to the already known Ki67 and disease stage. The improved patient stratification obtained using imaging parameters could have important clinical implications, such as for instance more aggressive treatment in patients with localized disease, but poor prognosis because of low SSTRd, or less intensive follow-up in patients with advanced disease, but likely good outcome owing to both low metabolic activity and high SSTRd.
